# Charting Shifts in Saccharomyces cerevisiae Gene Expression across Asynchronous Time Trajectories with Diffusion Maps

**DOI:** 10.1128/mBio.02345-21

**Published:** 2021-10-05

**Authors:** Taylor Reiter, Rachel Montpetit, Ron Runnebaum, C. Titus Brown, Ben Montpetit

**Affiliations:** a Food Science Graduate Group, University of California—Davis, Davis, California, USA; b Department of Viticulture and Enology, University of California—Davis, Davis, California, USA; c Department of Population Health and Reproduction, University of California—Davis, Davis, California, USA; d Department of Chemical Engineering, University of California—Davis, Davis, California, USA; University of Wisconsin—Madison; Harvard Medical School

**Keywords:** *Hanseniaspora uvarum*, *Saccharomyces cerevisiae*, diffusion mapping, environmental microbiology, fermentation, gene expression, transcriptional regulation

## Abstract

During fermentation, Saccharomyces cerevisiae metabolizes sugars and other nutrients to obtain energy for growth and survival, while also modulating these activities in response to cell-environment interactions. Here, differences in S. cerevisiae gene expression were explored over a time course of fermentation and used to differentiate fermentations, using Pinot noir grapes from 15 unique sites. Data analysis was complicated by the fact that the fermentations proceeded at different rates, making a direct comparison of time series gene expression data difficult with conventional differential expression tools. This led to the development of a novel approach combining diffusion mapping with continuous differential expression analysis (termed DMap-DE). Using this method, site-specific deviations in gene expression were identified, including changes in gene expression correlated with the non-*Saccharomyces* yeast Hanseniaspora uvarum, as well as initial nitrogen concentrations in grape musts. These results highlight novel relationships between site-specific variables and Saccharomyces cerevisiae gene expression that are linked to repeated fermentation outcomes. It was also demonstrated that DMap-DE can extract biologically relevant gene expression patterns from other contexts (e.g., hypoxic response of Saccharomyces cerevisiae) and offers advantages over other data dimensionality reduction approaches, indicating that DMap-DE offers a robust method for investigating asynchronous time series gene expression data.

## INTRODUCTION

During a wine fermentation, Saccharomyces cerevisiae metabolizes sugars and other nutrients to obtain energy for growth and survival, while also dealing with a common set of stresses caused by the must/wine environment. Given these general features of the system, the cellular activities of S. cerevisiae across wine fermentations are consistent, as reflected in a core gene expression program (CGEP) operating across fermentations (1–4). However, metabolism is not fixed, as S. cerevisiae dynamically responds to differences in the fermentation environment (e.g., nutrient levels, temperature, and varied microbial communities) to maintain cellular metabolism and overall fitness (1, 2, 5, 6). For example, differences in grape must nitrogen concentrations lead to changes in metabolism that result in altered aroma compounds in wine (7). This highlights the fact that metabolic adaptation to varied fermentation environments leads to differences in wine fermentation outcomes, including sensory variations. This relationship is mirrored by findings that show that genetic changes causing altered expression of select genes or pathways in S. cerevisiae lead to quantifiable differences in wine fermentation outcomes (8). These facts support the generally accepted idea that interactions between S. cerevisiae and the unique chemical and biological matrix of each grape must are central to defining primary fermentation characteristics. It is reasoned that these differences are the result of (i) the expression of unique genes outside those in the CGEP required for fermentation and/or (ii) the variation in expression of CGEP genes that changes the activity of various core pathways during fermentation.

The chemical and biological diversity of grape musts is due in part to biotic and abiotic pressures encountered by a grapevine during a growing season and the environmental interactions these pressures impart on different grape cultivars. For example, wines produced using genetically identical grapes under similar vinification conditions, but at different growing locations, have diverse sensory outcomes (9), many of which are reproducible across multiple vintages (10). After observing diverse sensory outcomes in wines where a consistent variable was vineyard location (9), quantifiable contributions of vineyard site were sought using S. cerevisiae gene expression as a biosensor to detect differences between fermentations. This was motivated by the fact that high-throughput gene expression surveys (microarray and RNA sequencing) have revealed the causes of stuck and sluggish fermentations (11), the triggers for entry into stationary phase (1, 2), and the impact of interspecies interactions on S. cerevisiae metabolism in wine (6, 12, 13). In addition, as an organism commonly used in life science and biotechnology research, the S. cerevisiae genome and transcriptome are well understood, with published data sets focused on gene expression in diverse environments, including wine (1–3, 14–16). This makes S. cerevisiae a powerful tool for understanding the wine fermentation environment and identifying key biotic and abiotic factors underlying fermentation outcomes.

Towards this end, time series RNA sequencing of Pinot noir fermentations was previously used to identify gene expression differences across 15 unique sites representing eight American Viticultural Areas (AVAs). However, using standard analysis methods (17–22), only the CGEP was identified across fermentations, not gene expression patterns indicating altered S. cerevisiae metabolism that would differentiate site (4). A major issue was that sampled fermentations proceeded at different rates, leading to asynchronous biological progression among sequenced samples with respect to fermentation progress (e.g., sugar consumption). This was problematic because samples need to be at the same stage of fermentation to interpret the biological significance of differentially expressed genes (3, 23). This is a common problem in time series experiments with multiple groups, and in some experimental systems, there are strategies to combat this issue (23). For example, in experiments that study the cell cycle, inhibitors arrest the cell cycle at the same stage across groups, thereby enabling comparisons (24).

To address a similar issue, methods have recently been developed for the analysis of single-cell RNA sequencing data from differentiating cells. In these experiments, as cells differentiate, absolute time may not reflect the extent of differentiation in each cell. Consequently, pseudotime analysis has been used to reorder cells from absolute time to the stage in differentiation relative to other cells undergoing the same process (25). In particular, diffusion maps have been used to reorder asynchronous cell populations because this analysis approach preserves relationships between samples (25). In general, diffusion mapping is a manifold learning technique that uses information from the *k* most similar samples to construct nonlinear composites of the major sources of variation among samples (26, 27). As a dimensionality reduction algorithm, diffusion maps extract latent variables that are inferred from relationships in the data, which can be used to represent composite sources of variation between samples.

Here, diffusion mapping with continuous differential gene expression analysis, termed DMap-DE, is used to analyze time series RNA-sequencing data from S. cerevisiae during hypoxia and fermentation. Diffusion maps were used to synchronize gene expression across treatment groups and to extract latent variables, termed diffusion components (DCs), which represent the dominant sources of structure in the data. Diffusion maps *per se* provide no suggestion of the underlying genes that lead to separation of samples along diffusion components; therefore, continuous differential expression analysis was performed using each diffusion component to determine what genes vary among samples across a diffusion component. This approach captured gene expression changes that occurred when yeast transitioned from aerobic to anaerobic metabolism during hypoxia or progressed through a fermentation. These findings suggest that DMap-DE enables analysis of diverse asynchronous time series gene expression data, revealing biologically relevant differences in gene expression among groups. In the context of wine, DMap-DE extracted the CGEP across Pinot noir fermentations, in addition to distinguishing differences between fermentations that reflected differences in the grape musts (e.g., site). These findings offer important insights into variable wine fermentation and sensory outcomes driven by site-specific factors.

## RESULTS AND DISCUSSION

**FIG 1:**
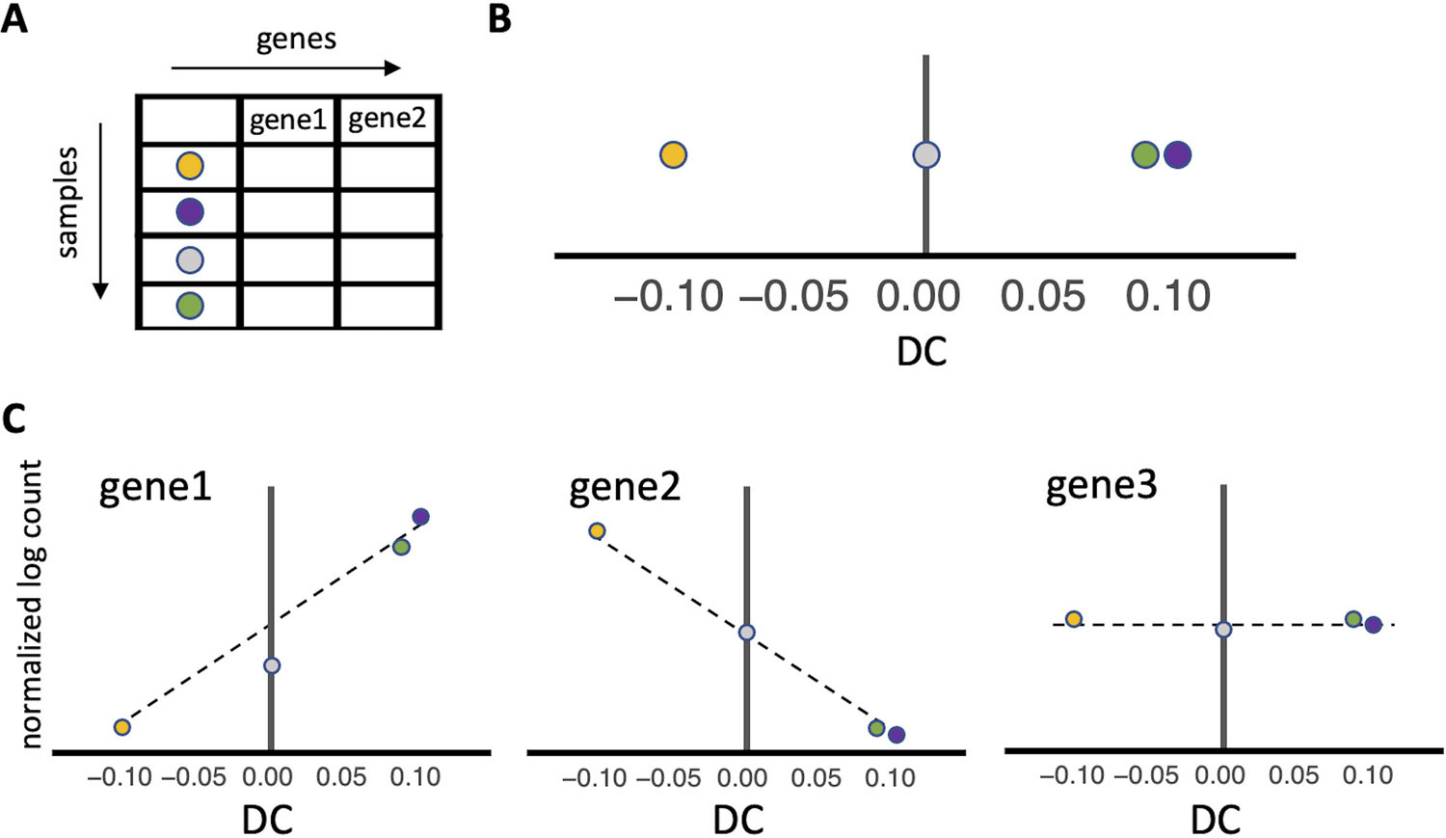
Extracting data from diffusion maps. Diffusion maps provide the underlying manifold in gene expression data through nonlinear dimensionality reduction. (A) When applied to many genes across many samples, diffusion maps extract features that represent combinations of genes that drive similarities and differences among samples. (B) The extracted features are termed diffusion components. Samples at either extreme of the diffusion component are the most different from each other, while samples that fall at the origin are invariant along that component. In the above graphic, the orange and purple dots are the most different, while the purple and green dots are the most similar. The gray dot lands at the origin and represents a sample that is not differentiated along the diffusion component (DC). Diffusion maps do not provide information on which genes lead to separation of samples along each diffusion component. (C) Performing differential expression using the diffusion component as a continuous variable reveals the genes that significantly contribute to separation of samples. In the graphic, gene 1 has significantly higher expression in samples that fall on the right extrema of the diffusion component (DC), compared to samples that fall on the left extrema resulting in a calculated positive log_2_ fold change (dashed line) along the diffusion component. Similarly, gene 2 has significantly lower expression in samples that fall on the right extrema of the diffusion component, compared to samples that fall on that left extrema, resulting in a calculated negative log_2_ fold change (dashed line) along the diffusion component. While all genes are used to perform differential expression, not all genes are differentially expressed along an individual diffusion component. In this example, gene 3 is not differentially expressed.

As a dimensionality reduction approach, diffusion maps reorder asynchronous cell populations while preserving relationships between samples to provide latent variables that reflect relationships between samples (25). We refer to these latent variables as diffusion components (DCs), the number of which is constrained by the number of samples in the data. Within each DC, a sample is represented by a single value, and samples that have similar underlying data (e.g., a similar gene expression profile) will have similar values (Fig. 1A and B). Moreover, samples at the origin of a DC (i.e., near 0) have gene expression profiles that do not vary along that component, while samples with positive or negative DC values diverge. Each DC captures diminishing structure among samples with the first diffusion component (DC1) accounting for the largest variation among all samples.

With the structure provided by diffusion mapping (i.e., values for each sample along a DC), continuous differential expression analysis can be used to identify genes with varied expression across a diffusion component (Fig. 1C). The calculated log_2_ fold change value for each gene corresponds to the change in gene expression for each unit change in the diffusion component value. Using this method, a positive log_2_ fold change value indicates the gene is expressed more highly in samples that segregate to the right extreme of a DC and is less expressed in samples that segregate to the left extreme of the DC. Conversely, a negative log_2_ fold change value indicates the gene is expressed more highly in samples that segregate to the left extreme of a DC and is less expressed in samples that segregate to the right extreme of the DC. It is important to reiterate that in this instance, a negative log_2_ fold change value does not indicate downregulation of expression of the gene. The combined use of diffusion mapping with continuous differential gene expression analysis (DMap-DE) is expected to identify changes in gene expression among samples that are linked to alterations in cellular metabolism over time or in response to the extracellular environment.

### Known gene expression changes during hypoxia are identified by DMap-DE.

**FIG 2:**
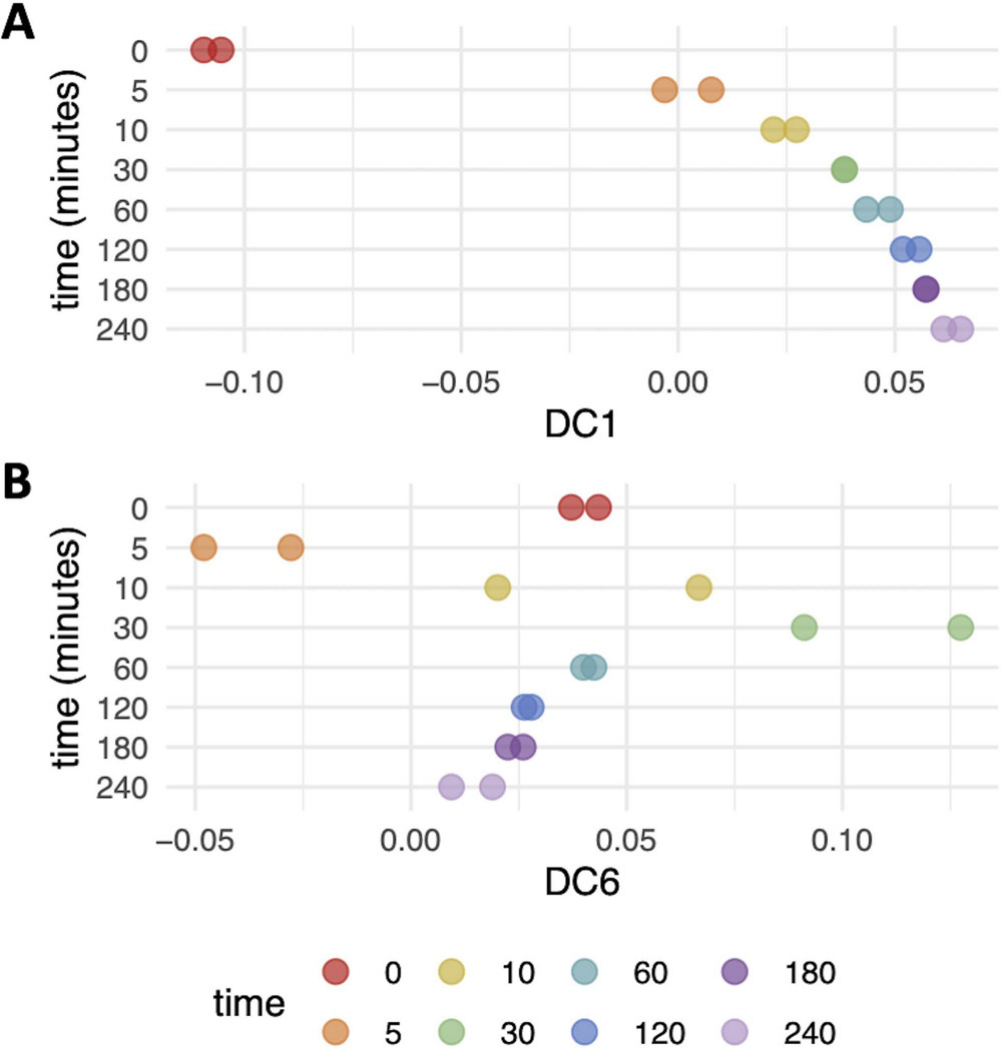
Diffusion mapping applied to S. cerevisiae exposed to nitrogen for 0 to 240 min. The trajectory of samples displayed along DC1 (A) captures the transition from aerobic to anaerobic metabolism, and that along DC6 (B) captures a transient transcriptome remodeling at 30 min.

To test the ability of DMap-DE to analyze and extract known changes in gene expression from RNA-sequencing data, a publicly available gene expression data set of S. cerevisiae during adaptation to hypoxia was identified (GEO accession no. GSE85595 and GSE115171). Hypoxia occurs when a cell becomes oxygen limited, which is accompanied by large-scale reprogramming of gene expression for continued growth (28). When DMap-DE was applied to this data set, an ordered time-dependent transition to a hypoxic phenotype along DC1 was observed (Fig. 2A; see Table S1 in the supplemental material). Sample positions along DC1 showed a rapid transition within 5 min of nitrogen exposure, indicating a fast metabolic transition to hypoxia that matured over the remainder of the time course. As part of this genetic reprogramming, a transient shift in gene expression was previously identified at ∼30 min of the hypoxic response and was shown to partially overlap the environmental stress response (28). Within the diffusion mapping data, this transient state at 30 min of hypoxia was observed in DC6 (Fig. 2B; and Table S1).

10.1128/mBio.02345-21.9 TABLE S1 Differential gene expression results for diffusion components DC1 and DC6 from hypoxia data. Download Table S1, XLSX file, 0.1 MB. Copyright © 2021 Reiter et al. 2021 Reiter et al. https://creativecommons.org/licenses/by/4.0/ This content is distributed under the terms of the Creative Commons Attribution 4.0 International license.

It was next investigated whether genes identified as being differentially expressed along DC1 (456 in total) matched oxygen-regulated genes identified by previous studies of hypoxia. Across seven microarray studies, 11 genes (3 aerobic, 8 hypoxic) were consistently identified as being involved in aerobiosis or anaerobiosis (compiled by Bendjilali et al. in reference 28). Along DC1, all 11 of these genes were identified as differentially expressed (*P* < 0.05). Similarly, applying DMap-DE to time series RNA-sequencing profiles of S. cerevisiae undergoing a hypoxic response (28), 239 of 291 (82.1%) aerobic genes were identified to be significantly expressed prior to exposure to nitrogen along DC1. In addition, 422 of 519 (81.3%) hypoxic genes were significantly induced after prolonged exposure to nitrogen along DC1 (Table S1). Genes identified by DMap-DE prior to hypoxia were significantly enriched for ribosome biogenesis, oxidative phosphorylation, and the sterol metabolic process, while genes identified as induced after prolonged exposure to nitrogen were enriched for the oxidation reduction process, cell wall, glycogen metabolic process, and glycolysis/gluconeogenesis (see Fig. S1 in the supplemental material). These findings align well with knowledge of the hypoxic transition in yeast (28, 29). Moreover, these results indicate that diffusion mapping with differential gene expression analysis captured global changes in gene expression during the hypoxic shift, including transient gene expression states, providing proof of concept for this method.

10.1128/mBio.02345-21.1 FIG S1 Gene set enrichment for differentially expressed genes along DC1 during onset of hypoxia. All enriched categories with *P* < 0.05 after Bonferroni correction are shown. Pathways on the right side of the figure are induced in samples with a high diffusion component value, while pathways on the left of the figure are induced in samples with a low diffusion component value. Download FIG S1, TIF file, 1.3 MB. Copyright © 2021 Reiter et al. 2021 Reiter et al. https://creativecommons.org/licenses/by/4.0/ This content is distributed under the terms of the Creative Commons Attribution 4.0 International license.

### DMap-DE detects the global shift in gene expression during primary fermentation.

Previously, inoculated primary fermentations of genetically similar Pinot noir grapes grown in California and Oregon were performed over multiple vintages at the UC Davis Teaching and Research Winery (4, 9, 30, 31). In 2019, time course RNA sequencing data were collected with the aim of using S. cerevisiae gene expression as an indicator of similarities and differences across fermentations from 15 sites representing eight AVAs (see Fig. S2A and B in the supplemental material). Samples were taken at times approximately corresponding to cellular adaptation after inoculation (2 and 6 h), early growth phase (16 h), stationary phase (64 h), and end of fermentation (112 h). The initial grape musts varied in parameters like initial nitrogen, pH, malic acid, tartaric acid, non-*Saccharomyces* microbial profile, and elemental profile, while the final wines differed in volatile profiles and sensory characteristics (9, 30, 31). Given the variable inputs and sensory differences described for wines from these sites (9), it was expected that there would be detectable differences in S. cerevisiae gene expression that would include genes known to impact the sensory outcome of wine (32). Yet, analysis of these data was only able to robustly identify the shared CGEP across fermentations (4). Site-specific differences were unable to be quantified because fermentations progressed at different rates, even with rigorous control of temperature at a 200-liter scale, leading to asynchronous biological progression among samples with respect to sampling time (Fig. S2C).

10.1128/mBio.02345-21.2 FIG S2 Locations of sites and sampling information across wine fermentations. (A) Map of sites used in this study within eight American Viticultural Areas (AVAs). (B) Primary fermentation sampling time points. Times are shown in hours and are relative to inoculation. (C) Site-specific fermentations across sampling time as measured by °Brix (total soluble solids, used as a proxy for sugar concentration). Note that figure panels A and C include locations and fermentation curves previously published for 10 sites (4), which are displayed together with five other sites also represented in this study. Download FIG S2, TIF file, 0.8 MB. Copyright © 2021 Reiter et al. 2021 Reiter et al. https://creativecommons.org/licenses/by/4.0/ This content is distributed under the terms of the Creative Commons Attribution 4.0 International license.

**FIG 3:**
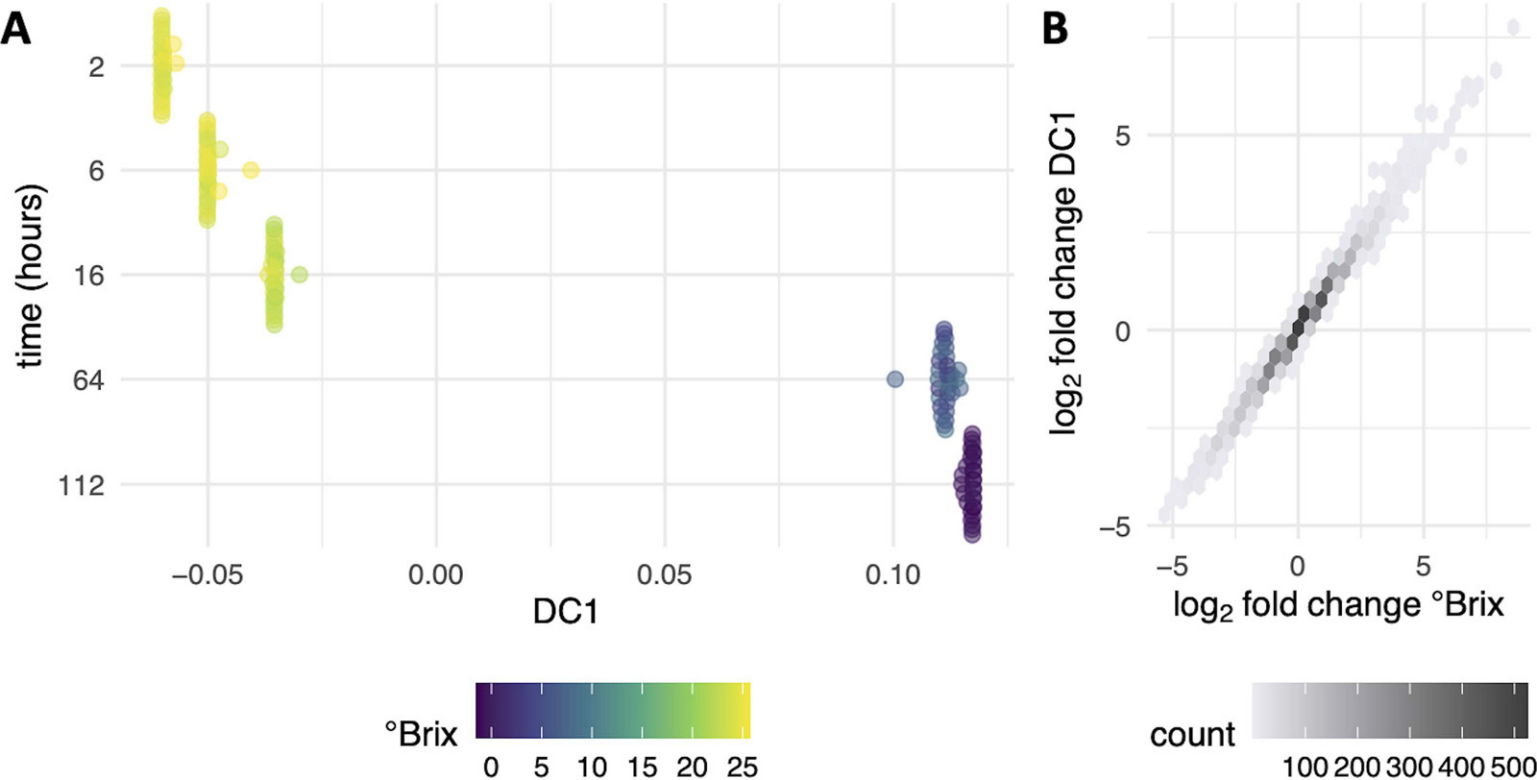
Diffusion mapping applied to S. cerevisiae during wine fermentation. (A) DC1 captures the metabolic transition that occurs as °Brix decreases during fermentation. Each point represents a sample from one time from one fermentation. Points that are closer along the *x* axis are more similar. The *y* axis is ordered by time, and points that occur at the same point on the *x* axis are arranged in a swarm for visualization. Points are colored by °Brix, a proxy for sugar concentration during fermentation, where °Brix = 0 indicates end of fermentation. (B) The graphic displays calculated correlations between differentially expressed genes in DC1 and genes that were previously determined to be differentially expressed as °Brix decreased, as detailed in reference 4.

To address this issue and gain insight into site-specific factors altering fermentation outcomes, DMap-DE was applied to the published sequencing data to identify gene expression patterns differentiating these fermentations. In DC1, which accounts for the largest variation among all samples, a clear transition during fermentation was observed by the ordering of samples across DC1 based on °Brix (Fig. 3A). To test whether DMap-DE captured the CGEP during fermentation along DC1, differential expression over DC1 was compared to values calculated previously across the °Brix variable using established methods of gene expression analysis (4). Log_2_ fold change values were strongly correlated between both methods of differential expression analysis (Fig. 3B), indicating that DC1 captured the global shift in gene expression during fermentation and DMap-DE identified the dominant gene expression signal (CGEP) as reported previously (4).

**FIG 4:**
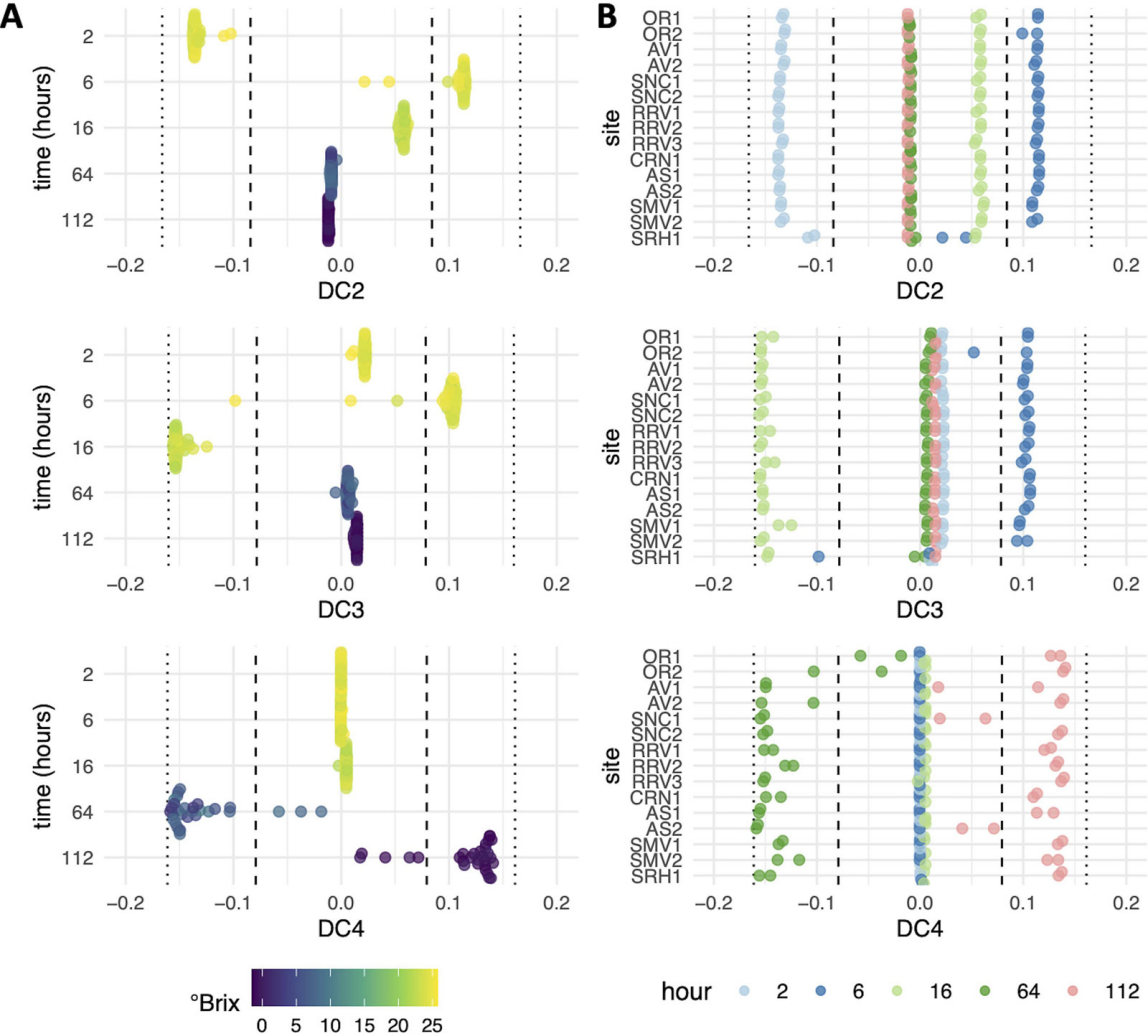
Samples in DC2 to DC4 separated by time in fermentation versus vineyard site. Plots are colored by °Brix (A) or hours postinoculation (B) and show that DC2, -3, and -4 capture different relationships among samples, with these components appearing to mainly capture shifts between stages of fermentation, not site. The vertical dashed and dotted lines in the graphs represent values that are 1 (dashed lines) or 2 (dotted lines) standard deviations from the mean.

**FIG 5:**
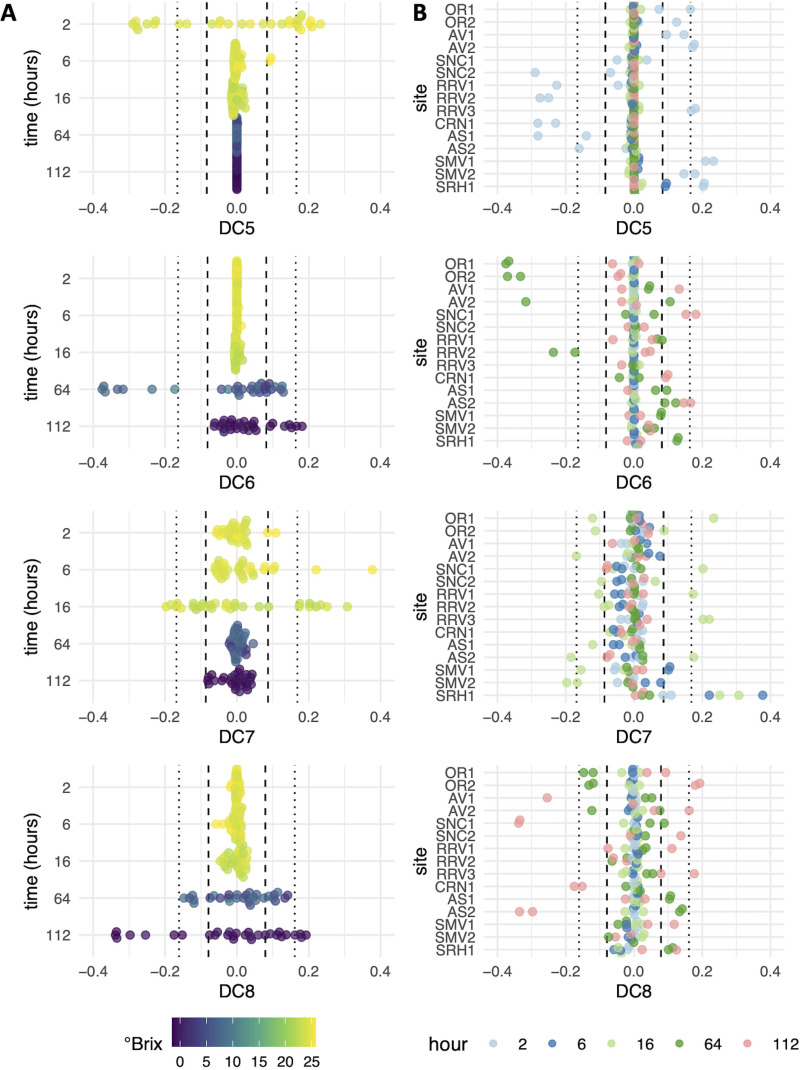
Samples in DC5 to DC8 separated by time in fermentation versus vineyard site. Plots are colored by °Brix (A) or hours postinoculation (B) and show that DC6, -7, and -8 capture differences between sites within the same stage of fermentation, as seen by samples clustering based on AVA, not °Brix. The vertical dashed and dotted vertical lines in the graphs represent values that are 1 (dashed lines) or 2 (dotted lines) standard deviations from the mean.

To identify less-dominant differences among samples, which may include site-specific differences, subsequent diffusion components (e.g., DC2 through DC8) were investigated. The specific patterns of gene expression across each DC are discussed below in detail, but in general, DC2 to DC4 organized samples with respect to the time of fermentation (Fig. 4), while samples from a time point or stage in fermentation separated across DC5 to DC8, demonstrating variation in gene expression among fermentations within a sampling time point (Fig. 5; see Table S2 in the supplemental material). As expected, given that each DC captures a diminishing structure among samples, the total number of differentially expressed genes also diminished as the diffusion component number increased (Table 1).

**TABLE 1:** Number of significantly differentially expressed genes for DC1 to -8

Diffusion component	No. of genes	
	Positive log_2_FC	Negative log_2_FC
DC1	470	457
DC2	169	16
DC3	32	293
DC4	57	74
DC5	13	0
DC6	92	46
DC7	50	7
DC8	0	24

10.1128/mBio.02345-21.10 TABLE S2 Differential gene expression results for each of the diffusion components DC1 to DC8 from fermentation data. Download Table S2, XLSX file, 0.4 MB. Copyright © 2021 Reiter et al. 2021 Reiter et al. https://creativecommons.org/licenses/by/4.0/ This content is distributed under the terms of the Creative Commons Attribution 4.0 International license.

### Lower diffusion components capture progression through fermentation.

The observed separation along DC2 to DC4 is based on °Brix levels and not site (compare Fig. 4A and B), with genes differentially expressed along these components indicating continued metabolic remodeling throughout fermentation (Table S2). Differences were specifically driven by cellular remodeling in early fermentation (DC2 and DC3) and starvation during late fermentation (DC4), based on the differentially expressed genes associated with each diffusion component (Table S2). Along DC2, there were clear separations among the 2-, 6-, and 16-h samples, while the 64- and 112-h samples fell on the origin (Fig. 4B). Within the genes captured along this component, the arginine biosynthetic process was enriched in genes that were more highly expressed in the 2-h samples (*ARG1*, *ARG3*, *ARG5* and -*6*, and *ARG8*) (see Fig. S3 and Table S2 in the supplemental material). Arginine is likely the most abundant amino acid in Pinot noir grape must (33), and genes that encode proteins involved in arginine biosynthesis are suppressed by the presence of arginine (34). Expression of these biosynthetic genes in early fermentation likely reflects that S. cerevisiae has yet to adapt to the wine environment by 2 h after inoculation. By 6 h of fermentation, expression of these genes decreased, potentially signaling completion of cellular adaptation to the grape must environment. Four of the 16 genes (*YMR244W*, *YPR078C*, *YGL117W*, and *YER085C*) differentially expressed in the 2-h samples have no known function. Given that very few genes were differentially expressed at 2 h, and they were enriched for arginine biosynthesis, one speculation is that these genes may have functions related to nitrogen and arginine biosynthetic processes. Alternatively, expression of these genes may be associated with other cellular processes for early adaptation to the must environment.

10.1128/mBio.02345-21.3 FIG S3 Gene set enrichment for differentially expressed genes along DC2 in the 2019 vintage. All enriched categories with *P* < 0.05 after Bonferroni correction are shown. Pathways on the right side of the figure are induced in samples with a high diffusion component value, while pathways on the left of the figure are induced in samples with a low diffusion component value. Download FIG S3, TIF file, 1.7 MB. Copyright © 2021 Reiter et al. 2021 Reiter et al. https://creativecommons.org/licenses/by/4.0/ This content is distributed under the terms of the Creative Commons Attribution 4.0 International license.

The 6-h samples segregated to the opposite extreme of DC2 and were the most differentiated from the 2-h samples along this component (Fig. 4B). Glycolysis was enriched among genes induced in these samples (Fig. S3 and Table S2) and was also accompanied by gene expression changes supporting transition to anaerobic metabolism. For example, induction of the anaerobic translation elongation factor encoded by *ANB1* was detected, which is optimally expressed below 0.5 μmol/liter O_2_ (35), likely indicating low must oxygen levels at this time point. Genes important for cell wall processes were also induced at 6 h, with *TIR1* to *-4* being 4 of the top 5 genes induced (Table S2). These genes encode cell wall mannoproteins required for anaerobic growth (36). These genes are also important in DC3 to separate the 6- and 16-h samples, along with many genes induced by anaerobiosis, including the *DAN1* and *PAU* genes (*PAU2* to -*5*, *PAU7*, *PAU8*, *PAU10* to -*12*, *PAU15* to -*17*, *PAU19*, *PAU20*, *PAU23*, and *PAU24*) in the 16-h samples (37, 38). Together, the induction of these genes regulated in response to oxygen across DC2 and DC3 likely signals the transition to anaerobiosis. In DC3, there were also many other biological processes, cellular compartments, and molecular functions enriched among the 293 genes that were induced in the 16-h samples (see Fig. S4 in the supplemental material), consistent with a transition to an active growth phase at this stage of fermentation. As diffusion components are ordered with the most variation among samples occurring first, DC2 and DC3 demonstrated that early metabolic remodeling was second only to larger gene expression changes that occur as °Brix decreases (e.g., captured in DC1) during fermentation.

10.1128/mBio.02345-21.4 FIG S4 Gene set enrichment for differentially expressed genes along DC3 in the 2019 vintage. All enriched categories with *P* < 0.05 after Bonferroni correction are shown. Pathways on the right side of the figure are induced in samples with a high diffusion component value, while pathways on the left of the figure are induced in samples with a low diffusion component value. Download FIG S4, TIF file, 1.9 MB. Copyright © 2021 Reiter et al. 2021 Reiter et al. https://creativecommons.org/licenses/by/4.0/ This content is distributed under the terms of the Creative Commons Attribution 4.0 International license.

Along DC4, separation of the 64-h samples from the 112-h samples was observed. In the 64-h samples, transmembrane transport, including amino acid and polyamine transport, were enriched categories among the genes that were induced (see Fig. S5 and Table S2 in the supplemental material). Induced genes (*DUR*3, *DAL5*, and *DAL7*) are involved in allantoin metabolism (39), which is a nonpreferred nitrogen source. Induction of these genes at 64 h likely indicates relief of nitrogen catabolite repression consistent with decreasing nitrogen concentrations and nutrient availability. Genes repressed by the presence of amino acids were also induced in the 112-h samples (*GAT2* and *ARG3*). *HXT13* and *MAN2* were also among the top induced genes, along with *HXT17*, in the 112-h samples (Table S2). These two *HXT* genes encode mannitol transporters, and *MAN2* encodes mannitol dehydrogenase (40). Expression of these genes would enable S. cerevisiae to metabolize mannitol as a nonpreferred carbon source (40–42). Mannitol is produced by non*-Saccharomyces* organisms, including lactic acid bacteria (43) and other non-*Saccharomyces* yeast (44). Expression of these genes late in fermentation could signal a switch to a metabolic program that utilizes nonpreferred carbon sources as the preferred sugars were exhausted. This could be tested in future vintages by measuring the concentrations of mannitol and other nonpreferred carbon sources in tandem with gene expression throughout fermentation.

10.1128/mBio.02345-21.5 FIG S5 Gene set enrichment for differentially expressed genes along DC4 in the 2019 vintage. All enriched categories with *P* < 0.05 after Bonferroni correction are shown. Pathways on the right side of the figure are induced in samples with a high diffusion component value, while pathways on the left of the figure are induced in samples with a low diffusion component value. Download FIG S5, TIF file, 0.8 MB. Copyright © 2021 Reiter et al. 2021 Reiter et al. https://creativecommons.org/licenses/by/4.0/ This content is distributed under the terms of the Creative Commons Attribution 4.0 International license.

Within lower diffusion components, outliers from select sites were also noted, which may indicate site-specific differences influencing S. cerevisiae gene expression during fermentation. For example, the 6-h samples from Santa Rita Hills site 1 (SRH1) were shifted toward 16-h samples along DC2 and DC3 (Fig. 4), potentially indicating faster cellular adaptation to the fermentation environment. However, fermentations from SRH sites proceeded at an average rate in the 2019 vintage, indicating gene expression differences did not impact the rate of fermentation (Fig. S2C). Another example involved a shift of the 64-h samples from Oregon site 1 (OR1) and OR2 along DC4 toward the 112-h cluster (Fig. 4), which may relate to nutrient conditions specific to OR sites (see further discussion below). Similarly, 112-h samples from Sonoma Coast site 1 (SNC1) and Arroyo Seco site 2 (AS2) shifted toward the 64-h samples (Fig. 4). Given that lower diffusion components separate samples by time in fermentation, it is expected that these outliers reflect differences between the musts (e.g., nutrient levels or presence of specific non*-Saccharomyces* organisms) that impact S. cerevisiae metabolism and the timing of gene expression transitions as fermentations progress.

Overall, the patterns of separation along DC1 to -4 reflect gene expression changes occurring as S. cerevisiae proceeds through fermentation, adapts to the increasingly nutrient-limited environment, and deals with associated stresses. While these changes appear common to the fermentations conducted here, additional work is required to address if individual processes captured in DC2 to -4 occur in the context of other wine strains and grape varieties or are unique to the wine yeast RC212 and Pinot noir fermentations. Nonetheless, these observations indicate that DMap-DE is a robust analysis approach for dealing with asynchronous gene expression data across fermentations. Moreover, the observations raise many questions about the genes important for defining separation along these DCs, including gene products involved in arginine, mannitol, and anaerobic metabolism. Of notable interest are the large family of *PAU* genes, the vast majority of which have no known function in S. cerevisiae, but have been previously noted as induced during fermentation and in response to stress (45).

### Higher diffusion components identify site-specific gene expression patterns.

The common patterns and existence of outliers across lower diffusion components indicate that information about specific sites was captured by these analyses. Because higher diffusion components were able to separate samples taken at the same time point (Fig. 5), gene expression differences across the higher DCs were used to investigate site-specific gene expression patterns (Table S2). In this way, S. cerevisiae activities specific to a site(s) can be inferred based on the gene expression patterns involved. Samples that separate to the extremes of each DC were focused on, as this separation indicates that these samples were the most differentiated at the transcriptome level.

At 2 h of fermentation, samples from Santa Maria Valley site 1 (SMV1), SRH1, Anderson Valley site 2 (AV2), and Russian River Valley site 3 (RRV3) fell 2 standard deviations above the mean along DC5, while samples from RRV2 and Carneros site 1 (CRN1) fell 2 standard deviations below the mean (Fig. 5). When comparing these sites, a standout difference was the induction of genes supporting vitamin metabolic and cell wall processes (see Fig. S6 and Table S2 in the supplemental material). Previous coculture experiments have demonstrated that S. cerevisiae induces genes involved in cell wall remodeling and vitamin biosynthesis in response to the presence of non-*Saccharomyces* yeasts (6, 46, 47). As such, the presence of non-*Saccharomyces* yeasts in the 2-h samples was correlated with DC5 values using gene counts for non-*Saccharomyces* yeasts determined for these fermentations in a previous study (30). Indeed, DC5 values correlated with total gene expression of Hanseniaspora uvarum (*R*^2^ = 0.49, *P* < 0.001), but not with total gene expression of other tested organisms (Table 2), suggesting that the presence of *H. uvarum* prior to these early fermentation samples may have impacted S. cerevisiae metabolism. This is consistent with a previous study, which reported that S. cerevisiae remodels its cell wall in the presence of *H. uvarum* at 3 h postinoculation in a wine fermentation (6). *PDC5* was among genes induced along DC5 in fermentations associated with *H. uvarum* (Table S2). *PDC5* encodes one of three isoforms of pyruvate decarboxylase, an enzyme involved in the formation of flavor-active higher alcohols in wine via the Ehrlich pathway (48–50). In wine fermentations, overexpression of *PDC5* has led to increased concentrations of 2,3-butanediol, other higher alcohols, and acetaldehyde (46, 49, 50). This suggests that the presence of *H. uvarum* may lead to gene expression changes directly impacting wine sensory outcomes. Given the potential for *H. uvarum* to impact S. cerevisiae gene expression and metabolism, it will be important to determine what factors promote *H. uvarum* (in)activity in select fermentations.

**TABLE 2:** Correlation between non-*Saccharomyces* organism total gene expression and DC5

Organism	*R* ^2^	*P* value
Aureobasidium pullulans	−0.03555	0.947
Botrytis cinerea	−0.03261	0.774
*Cladosporium* sp. strain SL 16	−0.03341	0.804
Hanseniaspora opuntiae	−0.03524	0.911
Hanseniaspora uvarum	0.490605	<0.001
Lachancea thermotolerans	−0.02741	0.638
Metschnikowia fructicola	0.069637	0.086
Pichia kudriavzevii	−0.02819	0.654
Rhizopus stolonifer	−0.02439	0.582

10.1128/mBio.02345-21.6 FIG S6 Gene set enrichment for differentially expressed genes along DC5 in the 2019 vintage. All enriched categories with *P* < 0.05 after Bonferroni correction are shown. Pathways on the right side of the figure are induced in samples with a high diffusion component value, while pathways on the left of the figure are induced in samples with a low diffusion component value. Download FIG S6, TIF file, 0.5 MB. Copyright © 2021 Reiter et al. 2021 Reiter et al. https://creativecommons.org/licenses/by/4.0/ This content is distributed under the terms of the Creative Commons Attribution 4.0 International license.

SMV and SRH are neighboring AVAs in southern California (Fig. S2A), and while samples from the SMV sites and SRH1 group together at 2 h, they separate at 16 h of fermentation along DC7 (Fig. 5). This suggests that while these sites were initially similar, they differed later in fermentation. While few genes were significantly induced in SMV versus SRH samples along DC7, *ADH4* was the top induced gene (Table S2). *ADH* genes encode alcohol dehydrogenases that play an important role in fermentation by facilitating transitions between acetaldehyde and ethanol involving the redox cofactor NAD^+^. *ADH1* encodes the primary alcohol dehydrogenase isoform responsible for this reaction during wine fermentation (51). Alcohol dehydrogenases are also involved in the formation of fusel alcohols within the Ehrlich pathway (52). As such, differences in *ADH4* gene expression could be an important site-specific difference with a role in S. cerevisiae metabolism and wine aroma development. Other genes more highly expressed in SMV sites were involved in cell growth processes, including translation (*MRP2* and *TIF2*), transcription (*MED1*), and cell division (*CLB6*) (Table S2). In site SRH1, more highly expressed genes along DC7 versus SMV were involved in oxidative stress (*RCK1*) and sporulation (*SPO74* and *SSP1*). These site-specific differences in gene expression involving factors linked to growth (SMV) versus stress (SRH1) indicate varied fermentation environments leading to altered gene expression at 16 h. Given that genes associated with the Ehrlich pathway and fusel alcohol anabolism differentiated SMV sites and SRH1 at 2 and 16 h of fermentation, the Ehrlich pathway may be an important component to consider in the context of site-specific differences in these Pinot noir wines.

**FIG 6:**
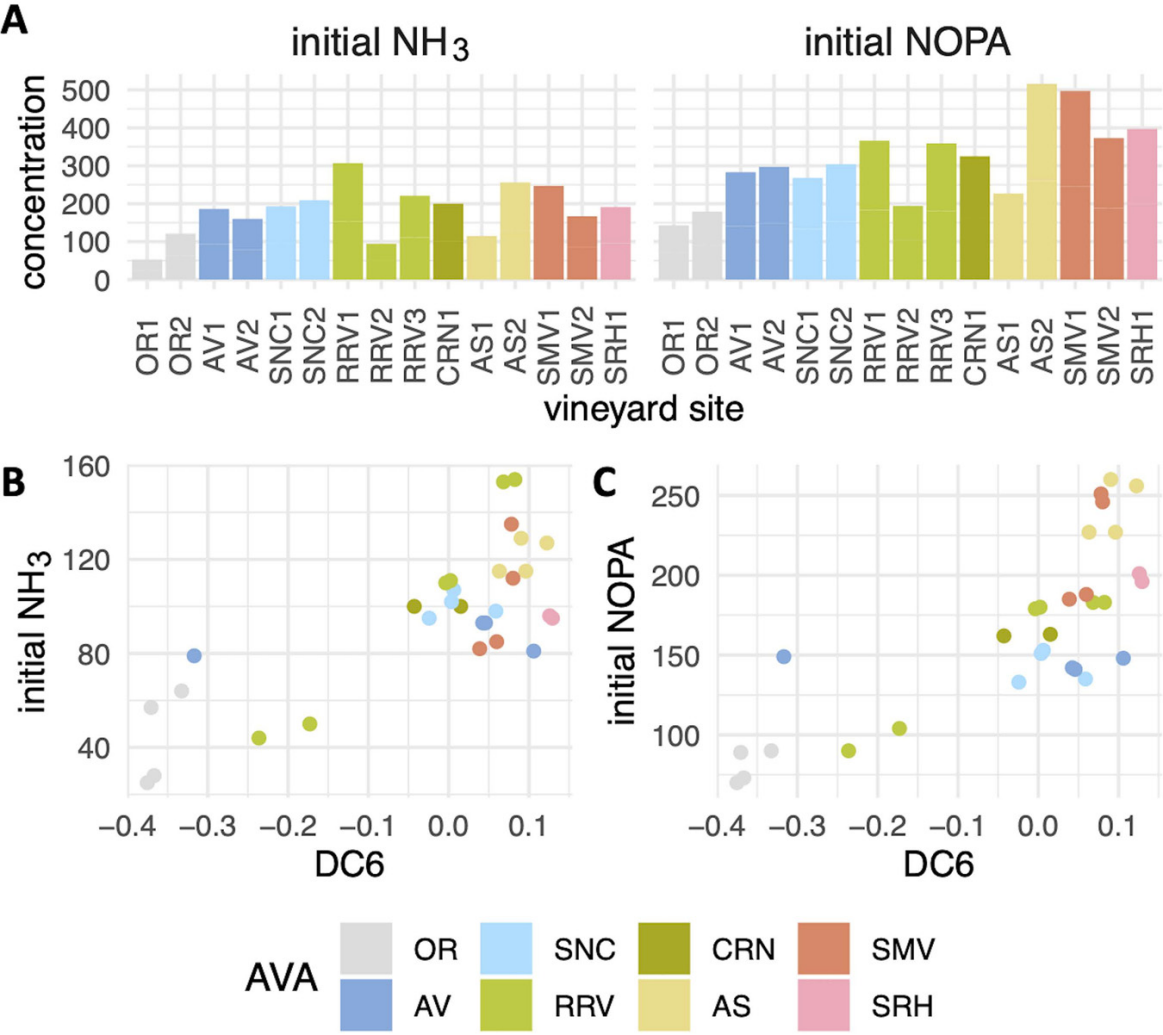
Initial nitrogen concentration in the grape must compared to DC6. (A) Initial concentration of NH_3_ and nitrogen by *o*-phthaldialdehyde assay (NOPA) across sites. (B and C) Initial NH_3_ (B) and NOPA (C) concentrations in grape must plotted against DC6 sample values. All concentrations are reported in mg/liter.

Separation was also observed among 64-h samples along DC6, with OR1/2 and RRV2 samples segregated to one extrema (Fig. 5). Genes induced in these samples were associated with nitrogen limitation (*DAL5*, *PUT1*, and *PUT2*) (1), while genes involved in ammonia metabolism (*MEP3*, *SSY1*, and *AUA1*) were induced in fermentations from sites at the other extrema (Table S2). In line with these patterns that reflect differences in nitrogen availability, DC6 values correlated with initial grape must nitrogen as measured by an *o*-phthaldialdehyde assay (NOPA) and NH_3_ measurements (initial NOPA, *R*^2^ = 0.62, *P* < 0.001; initial NH_3_, *R*^2^ = 0.60, *P* < 0.001), led by low initial nitrogen levels in OR1, OR2, and RRV2 (Fig. 6). While the initial nitrogen levels in OR1, OR2, and RRV2 were the lowest among all fermentations, these fermentations were supplemented approximately 24 h after inoculation with a combination of diammonium phosphate (DAP) and complex nitrogen sources to adjust total yeast assimilable nitrogen (YAN) levels to 250 mg/liter. Yet, these data indicate nitrogen limitation for these sites at 64 h, suggesting that the nitrogen additions may not have been sufficient to meet nutrient requirements in these fermentations. While it is also possible that initial nitrogen concentrations may correlate with DC6 for other reasons, these findings suggest that more research is needed to understand the impact of nitrogen additions on fermentation, including the timing of addition and the nitrogen source.

In DC8, SNC1 and AS2 separated at 112 h (Fig. 5): 14 of the 24 genes induced in these samples are of unknown function (Table S2). Among the induced genes with known functions were *DDR2* and *HSP30*, which are stress-related genes transcribed in response to a variety of environmental or physiological factors (53), as well as *YDL218W*, which is induced in response to the mycotoxin patulin produced by a variety of molds (54). Associated with these stress-related genes were genes that function in meiosis and sexual reproduction, including *SPO74*, *MFA1*, and *AFB1*. These data suggest that stresses in these fermentations could be driving the wine yeast into meiosis and a sexual reproduction cycle. This is of particular note, since the stresses associated with a wine fermentation environment are thought to impart strong selective pressures that drive adaptive evolution (55). This is reflected by the fact that S. cerevisiae strains associated with wine show a propensity for genetic diversity, including many instances of hybridization (56). Future research will be required to understand what particular stresses in Sonoma Coast site 1 (SNC1) and Arroyo Seco site 2 (AS2) are driving these unique patterns of gene expression, in addition to what outcome this has on fermentation performance.

Finally, across diffusion components, it is worth noting that fermentations from the same AVA were commonly grouped together, with diffusion component values within 1 standard deviation or less of other samples from the same AVA (Fig. 4 and 5). For example, fermentations from Oregon (OR in DC6 and DC8), Anderson Valley (AV in DC5 and DC8), and Santa Maria Valley (SMV in DC5) grouped together, providing support for the concept of the AVA and regional differences from the perspective of S. cerevisiae gene expression. However, we did not observe grouping among all fermentations from the same AVA along all diffusion components. For example, samples from Arroyo Seco (AS) grouped together along DC5 (2 h) and DC6 (64 h), but not in DC8 (112 h). The AS sites are separated by 1 km, and yet separation along DC8 suggests there was detectable variation in S. cerevisiae metabolism in primary fermentation (Fig. 4 and 5). Replicates from the same site have similar DC values, suggesting that lack of reproducibility in fermentations was not a factor in this observation. Similarly, fermentations from the Russian River Valley (RRV) did not group together along any diffusion component, suggesting that subappellations within the Russian River Valley are associated with significantly different S. cerevisiae gene expression patterns (Fig. 4 and 5). This matches recent findings that show subregional variation in elemental profiles of wine from the Russian River Valley (57). Importantly, the gene expression differences detected across each of these diffusion components provide candidate genes and pathways that may underlie site-specific fermentation outcomes.

### Comparison of diffusion maps to other dimensionality reduction techniques.

**FIG 7:**
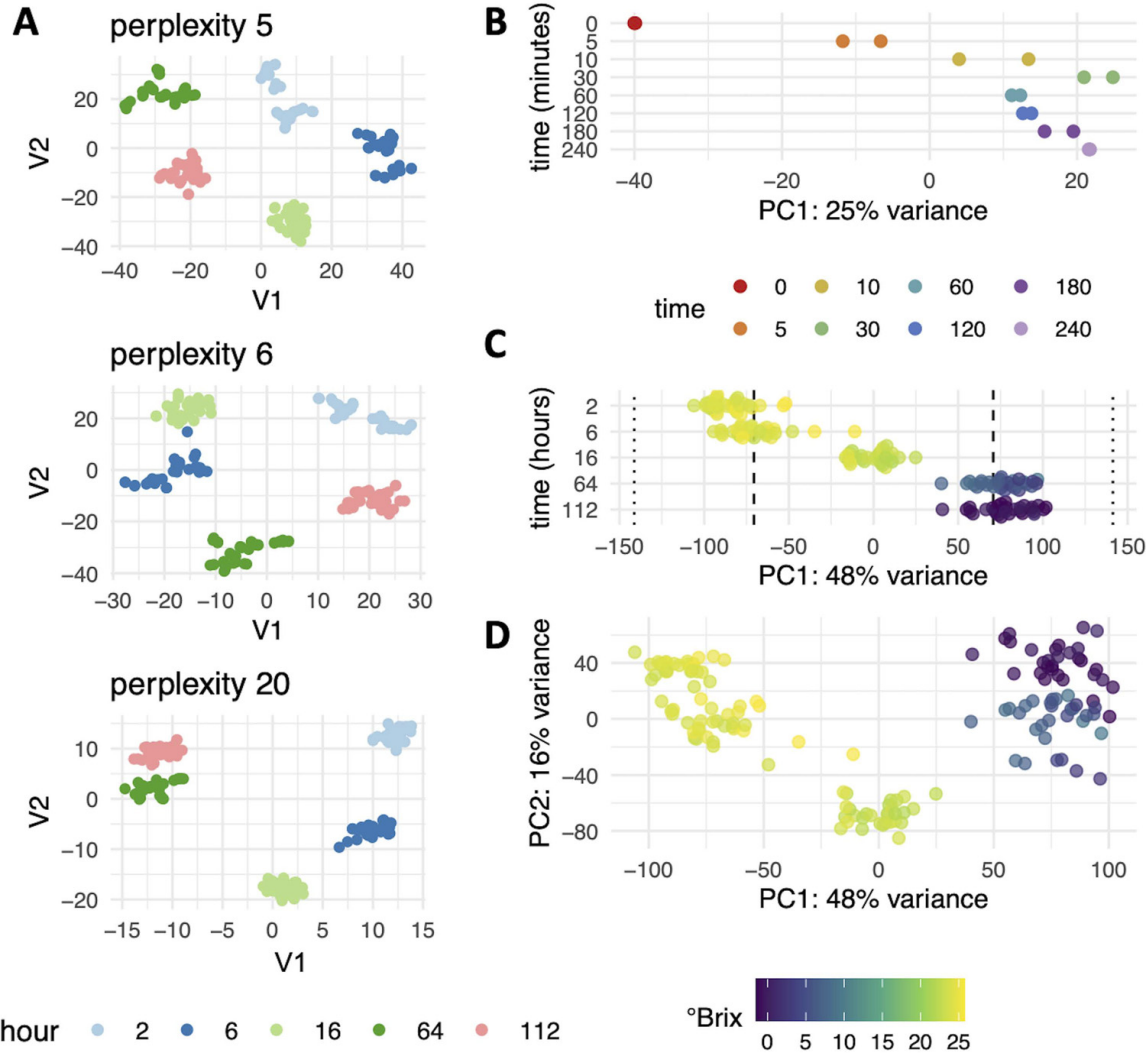
tSNE and PCA plots of wine and hypoxia data sets. (A) Perplexity is an internal parameter of tSNE plots that controls the balance between local and long-distance structure during tSNE computation. Plots of the same data at three different perplexity settings are shown with the impact on clustering and long-distance associations between clusters. Principal component 1 (PC1) values were plotted against time in fermentation (B) or time exposed to nitrogen (C). (D) Plot of PC1 versus PC2 using the wine data set, with samples colored based on °Brix.

The ability of DMap-DE to highlight gene expression patterns among the asynchronous samples tested here raises the question of how it compares to other methods. Generally, dimensionality reduction techniques are applied to RNA-sequencing data—often as a visualization method to detect outliers or cluster samples. Each algorithm produces a distinct reduced space accompanied by benefits and drawbacks (58, 59). In the context of differential expression, diffusion mapping has strengths over other dimensionality reduction algorithms like *t*-distributed stochastic neighbor embedding (tSNE) and principal-component analysis (PCA), which are commonly applied to sequencing data to identify sources of variation (58, 59). Unlike tSNE, diffusion mapping preserves long-distance structure between samples; tSNE excels at forming clusters by exaggerating local structure and thereby produces intuitive visualizations that demarcate groups, and as such, it is fundamentally inappropriate to use tSNE embeddings for continuous differential expression. This can be observed using different perplexities, a parameter within tSNE that controls the balance between local and global structure in the data when computing clusters. It can be observed that small changes in perplexity maintain the local clustering, while the relationship between clusters changes (Fig. 7A). This demonstrates that tSNE clusters samples taken at the same time point in fermentation but does not retain relationships between time points.

In comparison to PCA, diffusion mapping is less impacted by noise in gene expression data sets, producing a tighter grouping of similar samples within components and structure across components. For example, in both the fermentation and hypoxia data sets, DMap-DE sequentially orders samples by time along the first diffusion component, while PCA does not (Fig. 7B and C). In the fermentation data set, it is observed that higher principal components lack discernible patterns, separating samples neither by sampling time or site (see Fig. S7 and S8 in the supplemental material). Furthermore, diffusion mapping does not suffer from the “horseshoe effect,” a U-shaped positioning of samples in dimensionality reduction space that arises because of difficulty in discriminating differences between samples that share few expressed genes in common (60). Because diffusion mapping compares each sample to the *k* most similar samples, it avoids this effect, which was observed with PCA using fermentation data (Fig. 7D) and which can be observed in the PCA performed on the hypoxia data set in reference 28. These properties make diffusion mapping paired with differential gene expression analysis a powerful tool for interrogating time series gene expression data, as demonstrated here for S. cerevisiae in the context of the wine fermentation environment.

10.1128/mBio.02345-21.7 FIG S7 PC1 to PC4 separated by time in fermentation or vineyard site. Shown are sample positions across PC1 to PC4 as a function of time (A) or site (B) with plots colored by °Brix (A) or hours postinoculation (B). Download FIG S7, TIF file, 2.2 MB. Copyright © 2021 Reiter et al. 2021 Reiter et al. https://creativecommons.org/licenses/by/4.0/ This content is distributed under the terms of the Creative Commons Attribution 4.0 International license.

10.1128/mBio.02345-21.8 FIG S8 PC5 to PC8 separated by time in fermentation or vineyard site. Shown are sample positions across PC5 to PC8 as a function of time (A) or site (B) with plots colored by °Brix (A) or hours postinoculation (B). Download FIG S8, TIF file, 2.2 MB. Copyright © 2021 Reiter et al. 2021 Reiter et al. https://creativecommons.org/licenses/by/4.0/ This content is distributed under the terms of the Creative Commons Attribution 4.0 International license.

### Conclusions.

In this study, diffusion mapping was paired with differential expression to capture global shifts in gene expression. The DMap-DE method revealed differences in primary fermentation of Pinot noir wine from 15 sites, as well as changes in S. cerevisiae gene expression induced by hypoxia. Use of diffusion mapping was especially well suited for these data sets because, in both cases, cells progressed asynchronously through transcriptome changes with respect to sampling time. Through the analysis of wine fermentations, site-specific gene expression patterns correlating with *H. uvarum* gene expression and initial nitrogen composition of grape must were discovered, as well as indications of sexual reproduction in select fermentations. Together, these data provide important insights into the wine fermentation environment, including metabolic pathways, individual genes, and environmental factors that should be considered in the context of differential fermentation outcomes.

Given the tremendous complexity of gene-environment interactions, it is expected these data also serve to highlight the large amount of work to be done to understand both the biological mechanisms at play and how this knowledge can be applied by industry. Of particular note is the observed transcriptomic heterogeneity that arises from the same strain of yeast, fermented in the same facility, using grape must from genetically identical grape clones. How this variability changes across the diverse landscape of wine yeast strains and fermentation environments (e.g., grape varieties, including rootstocks, and associated chemical and microbiological profiles) remains to be seen. Importantly, the approaches pioneered here for studying S. cerevisiae gene expression in a complex environment using DMap-DE provide an effective tool to probe these questions.

## MATERIALS AND METHODS

### Sampling, sequencing, and preprocessing of wine fermentation sequencing samples.

The winemaking protocol (9, 31) and wine sample collection, RNA extraction, and sequencing (4, 30) have been described previously.

Sequencing data were downloaded from the Sequence Read Archive using accession no. PRJNA680606. Sequencing samples were preprocessed according to the manufacturer’s recommendations. First, we hard-trimmed the first 12 bp from each read and removed Illumina TruSeq adapters and poly(A) tails. Next, STAR was used to align reads against S. cerevisiae S288C genome (R64, GCF_000146045.2) with the following parameters: –outFilterType BySJout –outFilterMultimapNmax 20 –alignSJoverhangMin 8 –alignSJDBoverhangMin 1 –outFilterMismatchNmax 999 –outFilterMismatchNoverLmax 0.6 –alignIntronMin 20 –alignIntronMax 1000000 –alignMatesGapMax 1000000 –outSAMattributes NH HI NM MD –outSAMtype BAM SortedByCoordinate (61). UMI-tools was used to deduplicate alignments (62). Reads mapping to each open reading frame were quantified using htseq count (63).

### Hypoxia data set.

Gene expression count data were downloaded from GEO using accession no. GSE85595 and GSE115171.

### Construction of diffusion maps.

Diffusion maps were built as described previously (64). To build diffusion maps from wine fermentation samples, *k* = 10 nearest samples was used, while for hypoxia, *k* = 20 was used. We increased the *k* size for hypoxia given the larger number of samples (*n* = 150 in 2019 vintage and *n* = 336 in hypoxia). Prior to diffusion map construction, gene counts were to nonmitochondrial mRNA, and read counts were normalized based on total number of reads per sample (library size).

### Differential expression.

To determine which genes drove separation of samples along each component, differential expression was used to correlate each gene with diffusion component values. The R package limma was used to fit a linear regression model to each gene (20). As input to differential expression, raw sequencing counts were used as input to differential expression and were filtered and normalized with the limma package using the calcNormFactors() function (20). Using this model, the log_2_ fold change is the slope of the line for each unit increase in the diffusion component. Log_2_ fold change values were normalized by calculating the length of the diffusion component and multiplying all log_2_ fold change values by this amount: (maximum − minimum) × log_2_ fold change. Log_2_ fold change values that were greater than 2 were analyzed: i.e., genes with a log_2_ fold change of at least 2 between the most-separated samples along a diffusion component. Gene Ontology and KEGG enrichment analyses were performed using the functions enrichKEGG and enrichGO in the R clusterProfiler package to perform overrepresentation analysis (65). Bonferroni *P* value correction was performed, and *P* < 0.05 was used for the significance cutoff.

### Data availability.

Analysis code is available at https://github.com/montpetitlab/Reiter_et_al_2020_DiffusionMapping.
